# Assessing medically unexplained symptoms: evaluation of a shortened version of the SOMS for use in primary care

**DOI:** 10.1186/1471-244X-10-34

**Published:** 2010-05-17

**Authors:** Cristina Fabião, MC Silva, António Barbosa, Manuela Fleming, Winfried Rief

**Affiliations:** 1 Psychology Course. Department of Philosophy, Regional Centre of Portuguese Catholic University, Braga, Largo da Faculdade de Filosofia, 1, 4710 Braga Portugal; 2 Department of Population Studies, Institute for Biomedical Sciences Abel Salazar (ICBAS), University of Porto, Largo Abel Salazar, 2, 4099-003 Porto Portugal; 3 Centre of Bioethics, School of Medicine, University of Lisbon, Av. Prof. Egas Moniz, 1649-028 Lisboa Portugal; 4 Department of Behavioural Sciences, Institute for Biomedical Sciences Abel Salazar (ICBAS), University of Porto, Largo Abel Salazar, 2, 4099-003 Porto Portugal; 5 Department of Clinical Psychology and Psychotherapy, Philipps University of Marburg, Gutenbergstrasse 18, 35032 Marburg Germany

## Abstract

**Background:**

To investigate the validity and stability of a Portuguese version for the Screening for Somatoform Symptoms-2 (SOMS-2) in primary care (PC) settings.

**Methods:**

An adapted version of the SOMS-2 was filled in by persons attending a PC unit. All medically unexplained symptoms were further ascertained in a clinical interview and by contacting the patient's physicians and examining medical records, attaining a final clinical symptom evaluation (FCSE). An interview yielded the diagnosis of Clinical Somatization (CS) and the diagnosis of current depressive and anxiety disorders.

**Results:**

From the eligible subjects, 167 agreed to participate and 34.1% of them were diagnosed with somatization. The correlation between the number of self-reported and FCSE symptoms was 0.63. After excluding symptoms with low frequency, low discriminative power and not correlated with the overall scale, 29 were retained in the final version. A cut-off of 4 symptoms gave a sensitivity of 86.0% and a specificity of 95.5% on the FCSE and 56.1% and 93.6% at self-report. Stability in the number of symptoms after 6 months was good (k = 0.57).

**Conclusions:**

The 29 symptoms version of the SOMS-2 with a cut-off of 4 showed a high specificity and sensitivity, being reliable as a referral tool for further specialized diagnosis.

## Background

As the DSM (III-R and IV) Somatization Disorder was extremely rare in PC settings, and many patients presented medically unexplained symptoms (MUS), the need for an abridged somatization definition became evident. The Somatic Symptom Index (SSI 6/4) [1] and the Multisomatoform Disorder (MSD) [2] were proposed and validated. Several studies reported high prevalence estimates for Somatoform Disorders (SFD) in a PC context, notwithstanding the problems raised by different criteria and classification systems. Using ICD-10 criteria, values of 35.9% (current) and 39.4% (lifetime) were obtained [3]; with the DSM-IV criteria, 16.1% (current) [4] and 28.8% [5]; 19.7% with the SSI 6/4 criteria [6] and 8% with other abridged criteria [2,7].

Some researchers claim that screening for each symptom requires a clinical analysis as a sound basis for excluding organic disease or known "pathophysiological mechanisms" [8-10]. Other studies do not support the need for differentiation between explained and unexplained symptoms during the screening process [11,12].

Another dimension faced when screening Somatization is the heterogeneity of its clinical forms [13] and the attributional style [13]. The SOMS-2 [14,15] is a list of 53 physical complaints designed as a screener for SFD and "resembles a questionnaire version of the criteria for SFD according to the current classification systems" [16]. However an inconsistent recall of symptoms [17] has made SFD diagnoses according to current classification systems questionable. Evidence from GP evaluation and medical records is relevant in addressing a further challenge for the ascertainment of SFD patients according to current time criteria: the persistence over time of the tendency to present several somatoform symptoms.

Until now three studies have proposed screening tools for SFD in Portuguese. One used the ICD-10 Somatoform Disorders Symptom Checklist [18]; the second used the Questionnaire for physical manifestations of discomfort (Questionário de Manifestações Físicas de Mal Estar (QMFME)), based on the Psychosomatic Symptom Checklist (SUNYA) (Attanasio *et al*, 1984) to assess somatoform symptoms [19]. The QMFME included 19 items related to Nervous, Muscular, Digestive and Respiratory Systems and showed a satisfactory internal consistency within the four dimensions and as a whole. However the validation study used a reduced list of symptoms, and it is possible that symptoms with good discriminative power were left aside. The construct validity was not verified against Depressive and Anxiety Disorders and no attempt was made to validate criteria for clinical diagnosis [20]. A third study [21] explored Portuguese translations of the PILL (Pennebaker Inventory of Limbic Languidness, 1982), an inventory with 54 items, and the somatization subscale of the SCL-90-R. The last two tools presented good psychometric properties but were not validated against results of an interview and ask for physical symptoms in general, not for medically unexplained ones. Again the validation did not explore specificity in relation to anxiety or depressive disorders.

The Somatoform Dissociation Questionnaire-20 (SDQ-20) [22] was also validated in Portuguese. However it was designed to measure somatoform dissociation and dissociative disorders and not somatization in general.

This study was designed to validate the Portuguese version of the SOMS-2 for use in PC settings. The performance of the screening tool was also tested in the presence of specific comorbidity patterns.

## Methods

### Subjects and data collection

A PC unit with eight general practitioners (GPs) who provide care for a population of about 11,000 inhabitants was selected for this study. During a 10-day period alternating mornings and afternoons (August-September, 2007) all registered persons aged 18 years and older, able to read Portuguese, with no dementia, acute psychosis or mental retardation attending this unit were invited to participate. All willing persons had to fill in a socio-demographic questionnaire and the adapted Portuguese version of SOMS-2 [23,24], helped whenever necessary by psychology students. Within a short-time period participants were interviewed at the PC unit by a psychiatrist (CF), to ensure the somatoform nature of the symptoms reported and standardization of the severity/disability criteria. At the same time persons reporting no symptoms were again asked about SOMS-2 symptoms using a randomized order, for disclosure of false negatives at self-report. The same interviewer conducted the Portuguese validated version (5.0.0) of the MINI (1999, unpublished data displayed by Levy, P.). A trained assistant present at the interview, selected participants that, throughout the interview definitely did not accept medically unexplained causes for their symptoms: they were labeled probable "true somatizers" [13]. During a following two-month period all symptoms for which a medical explanation was in doubt were further discussed with the participant's GP. The history of somatoform complaints was made for each subject. As a standard procedure, medical records were always consulted, as well as other sources of medical information (if necessary) available on-line (hospital inpatient and outpatient contacts). Data from post-surgery diagnoses were obtained by contacting the surgeon in charge. Based on all information collected a final consensus about the symptom was reached (explained or unexplained), yielding the Final Clinical Symptom Evaluation (FCSE). Whenever information was insufficient for a decision on a reported "borderline" symptom, it was considered explained. Unexplained symptoms grafted onto medical conditions were also admissible. Participants were considered to have a somatoform symptom at FCSE even when they had an effective treatment for a SFD, provided the diagnosis was made in the previous two years.

After a period of six months stability of CS was tested in a random sample of approximately 15% participants who filled in the SOMS-2 and were interviewed by another psychiatrist using the same procedure, blind to first observation.

All participants received a description of the study and signed written informed consent, according to the Code of Medical Ethics of the World Medical Association Declaration of Helsinki. The study was approved by the Regional Primary Care Authority (Northern Region Health Administration).

### Measures

In the first section of the original SOMS-2 participants are asked to report physical symptoms they have suffered, either temporarily or continuously in the previous two years, that significantly disturbed their well-being or their personal lifestyle and for which doctors had not found a clear cause. A list of 53 somatoform symptoms, 5 only for women and 1 for men, are described. These are the physical symptoms listed for the diagnosis of SFD according to DSM-IV-TR criteria and Somatoform Autonomic Dysfunction according to the ICD-10 criteria. The second section has 15 questions to assess disability, the number of consultations resulting from the symptoms, and inclusion/exclusion criteria for all SFD. The original version showed scores for sensitivity between 86% and 100% and a 85% specificity for SFD according to DSM-IV criteria, as well as a good test-retest reliability and correlation of the number of self-reported symptoms with the number of symptoms yielded by a structured interview [14]. In this study an adapted Portuguese version of the SOMS-2 is used including a list of 46 symptoms, 45 for women and 42 for men [23]. The symptoms excluded because they were seldom stated by primary care users (<5%) were: frequent diarrhea, anal leakage, frequent bowel movements, loss of tactile and pain sensation, blindness, seizures and continuous or frequent vomiting during pregnancy.

Data collected from clinical interviews, GP longitudinal assessment of the case and all data from medical records were evaluated to form a diagnosis of Clinical Somatizers (CS), taking into account the number of validated unexplained symptoms (usually 5 or more at FCSE) and resulting disability, recurrence and lifetime persistence and age of onset (criteria available from the authors on request). Seven out of the 8 GPs and the 2 psychiatrists who collected and discussed the data had more than 20 years of clinical experience. The CS diagnosis was the "gold standard" to estimate the discriminative power of each symptom in the SOMS-2 list as well as its cut-off point.

Current depression and current anxiety disorders were diagnosed using the MINI, a fully structured interview, yielding 17 Axis I diagnoses according to DSM-III-R/IV and ICD-10 criteria, whose validation studies yielded good psychometric properties [25]. From the Portuguese version (5.0.0), which generates DSM-IV diagnoses, questions for the following conditions were selected: major depression with or without melancholic characteristics, dysthymia, hypomania, mania, suicidal attempts history, generalized anxiety disorder, simple phobia, social phobia, agoraphobia with or without panic attacks, panic disorder, posttraumatic stress disorder, obsessive compulsive disorder, substance abuse and dependence disorders (alcohol, cannabis, etc).

### Statistics

The overall prevalence and the prevalence ratio (PR) for specific sample strata were calculated and the respective 95% confidence intervals are reported. For obtaining a congruent overall inventory, the 46-symptoms adapted SOMS-2 was validated using 3 cumulative criteria applied to the FCSE. Only symptoms present in more than 2.5% of participants, with good discriminative power, that is, the 95% confidence interval for the positive likelihood ratio (LR+) not including 1 (the symptom should be more common in somatizers than in non-somatizers - sensibility/(1-specificity) and correlated with the overall scale (r ≥ 0.20), were included in the final version. The McNemar test or binomial distribution was used to compare the "prevalence" of symptoms at self-report and after clinical validation. The cut-off point for the SOMS-2 was studied using the ROC curve and the overall test characteristics were calculated (sensitivity and specificity) at self-report and after clinical validation, as well as for specific sub-samples. The positive and negative predictive values were calculated assuming the prevalence of somatization in the sample. The number of symptoms reported was used to measure stability rather than the specific symptoms reported. The statistic k was calculated for a series of two by two cross-tabulation according to the number of symptoms reported at baseline and 6 months after (0, ≥1), (<2, ≥2), (<3, ≥3), ..., until (<8, ≥8). The SPSS version 16 was used for statistical analyses and a probability value of 0.05 was used as the limit for Type I error (wrongly rejecting the null hypothesis).

## Results

Of the 928 eligible subjects, 18% agreed to participate in the whole evaluation (questionnaires and structured interview). The response rate almost doubled for women (20.5% *vs*. 13.0%) and declined gradually with age, from 28.0% for persons aged 18 to 44 years to 7.5% for those 65 or over. The age distribution of men or women in the sample is not significantly different from that of persons registered in the PC unit, though more women than expected were enrolled (chi-square = 26.5, df = 1, p < 0.001). Actually participants were mainly women (74.3%) and age ranged from 18 to 78 years, on average 43.7 (sd = 14.9), slightly higher for men (46.9 vs. 41.8 years). Most participants were married (65.3%), 65.8% had 9 or more years of full time education, 60.5% were employed and 62.3% lived in households inhabited by two or more generations (Table 1). The distribution of socio-demographic characteristics was not significantly different among somatizers and non-somatizers. The number of symptoms reported by participants on the SOMS-2 ranged from 0 to 20; 37.1% yielded no symptoms, 25% of women reported more than 4 symptoms and 25% of men more than 3 symptoms.

**Table 1:** Sample characteristics and prevalence ratio for somatization

Characteristics	Somatizers (n = 57)	Non somatizers (n = 110)	All (n = 167)	Prevalence ratio	95% CI
Gender					
Women	82.5	70.0	74.3	1.63	0.91-2.93
Men	17.5	30.0	25.7		
Age (years)^†^	44.0 (12.4)	43.6 (16.0)	43.7 (14.9)		
<45	47.4	53.6	51.5		
≥45	52.6	46.4	48.5	1.18	0.77-1.80
Marital status					
Single	14.0	21.8	19.2		
Married	70.2	62.7	65.3	1.47	0.77-2.81
Divorced	14.0	10.9	12.0	1.60	0.72-3.58
Widowed	1.8	4.5	3.6	0.67	0.10-4.40
Years of full time education					
<4	5.3	9.1	7.8	0.90	0.30-2.75
4-8	33.3	22.7	26.3	1.69	0.92-3.11
9-12	42.1	39.1	40.1	1.40	0.77-2.56
>12	19.3	29.1	25.7		
Occupational status					
Employed	66.7	57.3	60.5		
Unemployed	17.5	14.5	15.6	1.02	0.59-1.77
Retired	14.0	19.1	17.4	0.73	0.39-1.39
Others	1.8	9.1	6.6	0.24	0.04-1.59
Household composition					
Single	5.3	8.2	7.2		
Couple	14.0	23.6	20.4	0.94	0.30-2.98
Couple with sons/parents	70.2	58.2	62.3	1.54	0.56-4.22
Others	10.5	10.0	10.2	1.41	0.44-4.56
Income (minimum wage)^‡^					
<1	8.9	10.2	9.8	1.06	0.43-2.60
1-3	73.2	67.6	69.5	1.22	0.69-2.17
>3	17.9	22.2	20.7		
Comorbidity					
Depression (current)	38.6	12.7	21.6	2.28	1.56-3.36
Anxiety (current)	38.6	15.5	23.4	2.06	1.39-3.06
Any current depression/anxiety	57.9	21.8	34.1	2.65	1.75-4.03
Personality disorder	24.6	9.1	14.4	1.94	1.27-2.95
Hypochondria	6.0	3.0	4.0	1.53	0.67-3.53
Medicated psychoses	1.8	1.8	1.8		
SOMS-2					
No of symptoms^§^					
women	5 [2-7]	1 [0-3]	2 [0-4]		
men	2 [0-11]	0 [0-2]	1 [0-3]		
Wellbeing affected^¶^	83.0	50.0	64.8	2.65	1.39-5.06
ADL affected^¶^	72.3	48.3	59.0	1.81	1.09-3.01

Prevalence ratio: prevalence in category indicated against category with no values shown; CI: confidence interval; ^†^mean (standard deviation); ^‡ ^for 164 participants; ^§^median [1^st ^and 3^rd ^quartiles]; ^¶^for those reporting symptoms (n = 105) and prevalence ratio for (yes vs. no)

The overall prevalence of CS was 34.1% (95%CI: 27.4-41.6) and 10 participants with CS were probable "true" somatizers. A current depressive disorder (CDD) was present in 21.6% of participants, and 19,2% had major depression, while a current anxiety disorder (CAD) was present in 39 (23.4%) participants; they overlap in 18 (10.8%) participants and 110 (65.9%) were free from depression and anxiety. Overall 48.5% of participants had CS, anxiety or depressive disorders. In 33 participants (19.8%) CS was comorbid with a depressive or anxiety DSM-IV Disorder. Twenty-two cases (13.2%) of CS had CDD and the same number had CAD. Twenty nine (80.6%) persons with CDD also had CAD or CS. Twenty nine (74.4%) persons with CAD also had at least one CDD or CS and 33 (57.9%) with CS also had a current depressive or anxiety disorder.

The prevalence of CS was higher in women than men (PR = 1.63) and in those with 4-8 years of education compared to those with 12 years or more, more than doubling in participants with any current depression/anxiety (PR = 2.65). The prevalence of CS is also higher in persons reporting that symptoms affected their well being or activities of daily living (Table 1).

**Figure 1:**
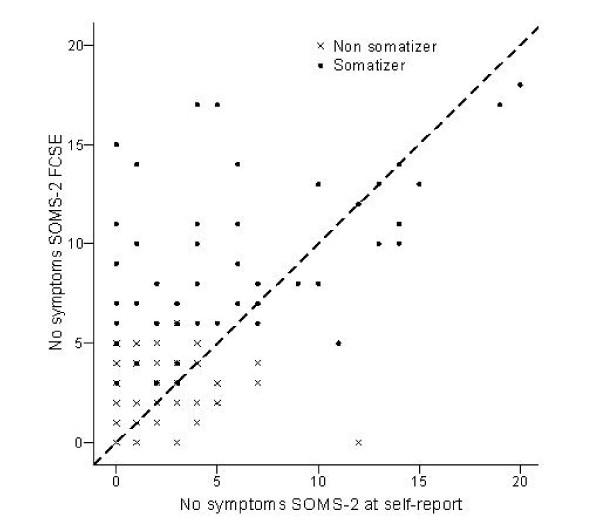
**Scattergram of No. of symptoms self-reported and clinically revised**. (FCSE: final clinical symptom evaluation).

The number of SOMS-2 symptoms emerging from the final clinical symptom evaluation (FCSE) ranged from 0 to 18 and 44 (21.4%) participants yielded no symptoms. The Spearman's rank correlation between the number of self-reported and FCSE symptoms was 0.63, higher in non somatizers (r = 0.58) than in somatizers (r = 0.42), these having on average 3 more symptoms at the FCSE (Figure 1).

### Soms-2 validation

#### 1. Frequency, discriminative power and internal consistency of symptoms

From the overall 46 symptoms evaluated at the FCSE in the 167 participants, 17 were excluded from the final inventory because they were stated by less than 2.5% of participants (7, 9, 38, 39, F52 and M53). Symptoms 16, 17, 18, 19, 33, 42, 44, F48, F49 and F50 were also excluded, since their discriminative power was low and symptom 3 was excluded since the correlation with the overall inventory was low (Table 2). Two symptoms were only present in somatizers, painful breathing and paralysis/weakness, indicating a high discriminative power, followed by nausea (30.8), difficulty in swallowing (28.9), bringing swallowed food up again (27.1), vomiting (21.2), sexual indifference (10.8), strong heart pounding (9.6), and amnesia (9.6). Correlation with the global scale was low for some "pain symptoms" (4, 5, 8) and high for strong heart pounding (0.54), sexual indifference (0.48), bloating or sweating (0.45) and burning sensations in chest or stomach (0.43). Most symptoms were underreported by participants, and 13 symptoms were more "prevalent" at the FCSE than when self-reported (1, 2, 6, 10, 12, 13, 15, 24, 25, 28, 29, 36, 46). Considering the reduced list of 29 symptoms, R-SOMS-2, the Spearman's rank correlation between self-report and FCSE was 0.63 for women and 0.67 for men, while the corresponding values for the 46 symptoms list was 0.62 both for men and women. The internal consistency evaluated by Cronbach's alpha was 0.83 for both FCSE and SOMS-2 at self report, while for the original 46 symptoms list the corresponding values were 0.82 and 0.85.

**Table 2:** Frequency of SOMS-2 symptoms (%), discriminative power (LR+) and correlation with SOMS-2 full scale

		SOMS-2 self reported			SOMS-2 FCSE			
			
	Symptoms	(%)	Positive Likelihood ) ratio (95% CI)		(%)	Positive Likelihood ratio (95% CI)		**r**_**pbi**_
1	Headaches	21.6	1.54	(0.8-2.7)	31.7	2.72	(1.7-4.2)	0.31
2	Pain in the stomach	6.6	2.30	(0.7-7.3)	18.6	4.70	(2.3-9.6)	0.37
4	Joint Pain	6.0	2.90	(0.8-9.8)	9.6	3.21	(1.2-8.4)	0.24
5	Pain in the legs and/or arms	11.4	2.65	(1.1-6.2)	12.0	2.36	(1.0-5.4)	0.21
6	Chest Pain	7.2	3.86	(1.2-12)	13.2	5.15	(2.1-12)	0.33
8	Pain during sexual intercourse	4.8	5.79	(1.2-28)	7.2	5.79	(1.6-20)	0.22
10	Nausea	5.4	2.41	(0.7-8.6)	10.2	30.80	(4.2-227)	0.23
11	Bloating	15.6	4.34	(2.0-9.4)	14.4	5.79	(2.4-14)	0.45
12	Discomfort in the area around the heart	5.4	6.75	(1.5-31)	10.2	6.27	(2.1-18)	0.36
13	Vomiting (pregnancy excluded)	3.0	5|57		7.2	21.20	(2.8-160)	0.32
14	Bringing swallowed food up again	5.4	15.40	(1.9-120)	9.0	27.10	(3.6-200)	0.37
15	Hiccough, or burning sensations in chest or stomach	6.6	5.15	(1.4-19)	12.0	5.79	(2.2-15)	0.43
22	Frequent urination	7.2	5.79	(1.6-20)	6.6	5.15	(1.4-18)	0.28
24	Strong heart pounding	8.4	11.58	(2.7-50)	18.0	9.65	(3.9-24)	0.54
25	Stomach discomfort or churning feeling in the stomach	13.8	2.51	(1.2-5.4)	23.4	3.86	(2.2-6.9)	0.39
26	Sweating (hot or cold)	12.0	4.50	(1.8-11)	14.4	7.33	(2.9-18)	0.45
27	Flushing or blushing	6.6	8.68	(1.9-39)	4.8	5.79	(1.2-27)	0.28
28	Breathlessness (without exertion)	3.6	9.65	(1.2-80)	11.4	7.24	(2.5-21)	0.33
29	Painful breathing or hyperventilation	2.4	5.79	(0.6-54)	6.0	10|57		0.38
30	Excessive tiredness or mild exertion	17.4	4.29	(2.1-8.8)	19.8	5.15	(2.5-10)	0.40
31	Blotchiness or discoloration of the skin	4.8	3.21	(0.8-13)	4.2	11.58	(1.4-93)	0.26
32	Sexual indifference (loss of libido)	7.8	23.20	(3.1-174)	10.8	15.43	(3.7-65)	0.48
34	Impaired coordination or balance	5.4	3.86	(1.0-15)	7.8	6.43	(1.8-22)	0.38
35	Paralysis or localized weakness	2.4	4|57		4.2	7|57		0.42
36	Difficulty swallowing or lump in the throat	6.0	17.4	(2.3-134)	9.6	28.95	(3.9-213)	0.39
37	Aphonia	6.0	4.50	(1.2-17)	7.2	5.79	(1.6-21)	0.25
41	Unpleasant numbness or tingling sensations	12.6	3.86	(1.7-9.0)	11.4	7.24	(2.5-21)	0.32
46	Amnesia (loss of memory)	10.2	3.54	(1.4-9.1)	14.4	9.65	(3.5-27)	0.40
47	Loss of consciousness	1.8	3|57		4.8	8|57		0.35
								
	Excluded (no discriminative power in FCSE)							
3	Back Pain	11.4	1.74	(0.7-4.0)	16.2	2.81	(1.4-5.6)	0.12
7	Pain in the anus	1.8	3.86	(0.4-41)	0.6	1|57		0.13
9	Pain during urination	1.2	2|57		1.2	2|57		0.23
16	Food intolerance	0.6	1|57		1.8	3.86	(0.4-41)	0.19
17	Loss of appetite	7.2	3.86	(1.2-12)	9.6	2.48	(0.9-6.3)	0.20
18	Bad taste in mouth, or excessive coated tongue	6.6	3.38	(1.0-11)	4.2	4.82	(0.9-24)	0.16
19	Dry mouth	11.4	2.65	(1.1-6.2)	7.2	1.38	(0.5-4.2)	0.05
33	Unpleasant sensations in or around the genitals	1.8	3.86	(0.4-42)	3.6	3.86	(0.7-20)	0.16
38	Urinary retention	0.0			0.0			
39	Hallucinations	1.8	3|57		1.2	2|57		0.10
42	Double vision	3.0	1.29	(0.2-7.5)	3.6	3.86	(0.7-20)	0.20
44	Deafness	1.8	3|57		2.4	5.79	(0.6-54)	0.08
F48	Painful menstruation	8.1	1.09	(0.3-3.7)	12.1	1.43	(0.6-3.7)	0.33
F49	Irregular menstruation	2.4	0.82	(0.1-8.8)	3.2	0.55	(0.1-5.1)	0.18
F50	Excessive menstrual bleeding	4.0	2.46	(0.4-14)	8.1	2.46	(0.7-8.3)	0.27
F52	Unusual or copious vaginal discharge	0.0			0.0			
M53	Erectile or ejaculatory dysfunction	2.3	1|10		2.3	1|10		0.15

CI: confidence interval; r_pbi_: point-biserial correlation coefficient with the overall sum of symptoms; F48 to F52: symptoms only for women; M53: symptom only for men; for these groups of symptoms frequencies, positive likelihood and correlations were calculated for the respective groups (124 women and 43 men); whenever the Positive Likelihood ratio [sensibility/(1-specificity)] was not defined the number of somatizers with that symptom is indicated (sensibility) since non-somatizers did not have the symptom (specificity = 1)

#### 2. Sensitivity and specificity (cut-off)

**Figure 2:**
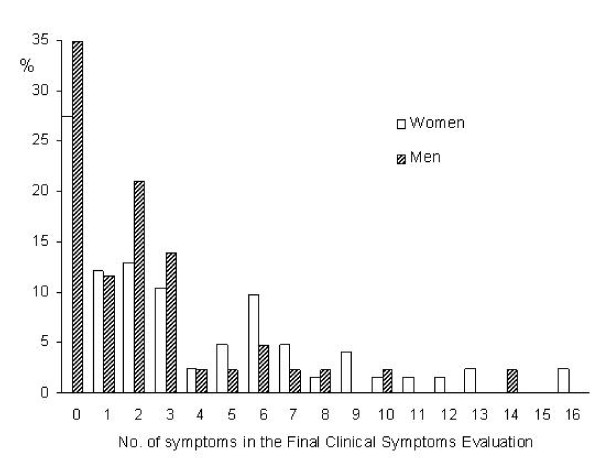
**No. of symptoms (out of 29) in women and men showing the "natural" 4 symptoms cut-off point**.

Figure 2 shows the "apparent" cut-off point of 4 symptoms in the SOMS-2 with 29 symptoms, calculated by the ROC curve. Table 3 shows the sensitivity and specificity for this cut-off on the 29 symptoms list as well as for the full list. The ROC curve for the FCSE yielded the cut-offs of 6 symptoms for women and 4 for men, but different cut-off for self-report, 4 and 5, respectively. An overall sensitivity of 86.0% and specificity of 95.5% was attained both for SOMS-2 and R-SOMS-2 at FCSE, and the corresponding values at self-report are 57.9% and 88.2% for the former, decreasing slightly the sensitivity and increasing the specificity for the R-SOMS-2 symptoms list, 56.1% and 93.6%. The positive and negative predictive values are PPV = 90.7% and NPV = 92.9% for FCSE and the corresponding values for the self-report are PPV = 71.7% and NPV = 80.2% for the SOMS-2 and PPV = 82.1% and NPV = 80.5% for the R-SOMS-2.

**Table 3:** Test characteristics (%) and predictive values (%) at self-report and FCSE

	SOMS-2 FCSE						SOMS-2 self-report				
			
Cut-off points/No symptoms	Sensitivity	Specificity	PPV		NPV		Sensitivity	Specificity	PPV		NPV
Women (≥6/45)	87.2	98.7	97.6		92.7	(≥4)	61.7	87.0	74.4		78.8
Men (≥4/42)	80.0	87.9	66.7		93.5	(≥5)	40.0	90.9	57.1		83.8
All	86.0	95.5	90.7		92.9		57.9	88.2	71.7		80.2
Women (≥4/29)	89.4	94.8	91.3		93.6		59.6	94.8	87.5		79.3
Men (≥4/29)	70.0	97.0	87.5		91.4		40.0	90.9	57.1		83.3
All	86.0	95.5	90.7		92.9		56.1	93.6	82.1		80.5

FCSE: Final Clinical Symptoms Evaluation; PPV: positive predictive value; NPV: negative predictive value

**Figure 3:**
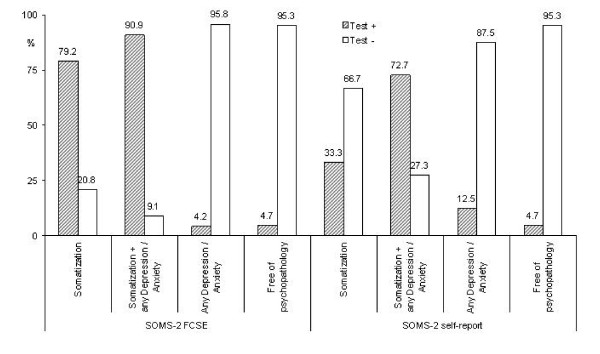
**Test characteristics (4 symptoms cut-off point) for different comorbidity patterns**. (somatizers, n = 24; somatizers with any depression/anxiety, n = 33; non-somatizers with any depression/anxiety, n = 24; free of psychopathology, n = 86).

Considering the frequency of comorbid conditions in the sample, CS with concomitant anxiety and/or depression, the test characteristics were also calculated considering four different groups: "pure" somatizers (somatization without any depression/anxiety), somatizers with any depression or anxiety and corresponding groups for non-somatizers (Figure 3). Sensitivity reaches 90.9% whenever somatizers have some depression and/or anxiety, though attaining a lower value, 79.2%, in "pure" somatizers. Specificity keeps an almost constant value of 95% for persons with some depression and/or anxiety or for non somatizers free from any depression/anxiety. At self-report it discloses only 33.3% of "pure somatizers" increasing its sensitivity whenever somatizers have some depression and/or anxiety (72.7%). The self-report R-SOMS-2 performs equally well as FCSE for somatizers without any depression or anxiety (95.3%).

#### 3. Stability (intra-subject variation)

The correlation between the number of symptoms reported at base-line on the full list of symptoms and after a 6-month period for a sample of 24 persons was r = 0.67, increasing to 0.69 in the R-SOMS-2. Considering the series of cut-off points from 1 to 8, the values of agreement (k) were respectively 0.63, 0.50, 0.52, 0.33, 0.33, 0.41, 0.47 and 0.65. Using the 29 symptoms list the values obtained were 0.55, 0.52, 0.52, 0.57, 0.48, 0.48, 0.43 and 0.50, respectively. Thus the cut-off of 4 symptoms has the best agreement in terms of the number of symptoms reported.

## Discussion

Both the SOMS-2 and the 29 items reduced version, R-SOMS-2, showed good characteristics in detecting CS, though dependent on target population and the presence of concomitant psychiatric diagnoses. As a screening tool for primary care settings, the R-SOMS-2 with a cut-off of four symptoms both at self-report and after clinical validation of symptoms reported, showed a high specificity (93.6%) and satisfactory sensitivity (56.1%). Whenever concomitant disorders such as anxiety and/or depression are present the sensitivity increases (72.7%) and specificity still keeps a high value (87.5%). Moreover the R-SOMS-2 showed a relatively high stability in the number of self-reported symptoms over a 6-month period (k = 0.57). According to the actual prevalence found in this study (34.1%) it showed also high positive and negative predictive values, 82.1% and 80.5%, respectively. On the other hand the adapted SOMS-2 Portuguese version showed both a high internal consistency (0.85) and a slightly higher sensitivity (57.9%) at self-report. After clinical validation sensitivity and specificity were equal for the two SOMS-2 versions, 86.0% and 95.5%, respectively.

It is possible that by strictly instructing the participants to be aware of severity criteria, the common underestimation of somatoform symptoms [10] has been reinforced in our study. Although stability of single symptoms or disorders is not satisfactory [17,26], stability of somatization seems to be less problematic when symptoms are grouped into syndromes [26,27]. Correlation between self-reported symptoms and symptoms assessed by doctors (with regard to the previous two years) was 0.63, lower than the 0.75 reported by Rief and colleagues [18]. The R-SOMS-2 includes not just the most frequent symptoms in PC, but rather those more frequently positive among somatizers in comparison to non-somatizers, that is, with high discriminative power and with a reasonable high correlation with the total scale. It may seem unreasonable excluding symptoms with relatively high frequency, such as "dry mouth" or "painful menstruation", but they are not "characteristic" of CS, they are as well important in other patients, therefore not adequate for discriminating CS. On the other hand a quite frequent symptom like "back pain" was also excluded because the correlation with the overall scale was low, meaning that its pattern of variation did not follow the overall scale pattern. The three cumulative criteria for including symptoms in this revised version resulted in a shorter scale with properties similar to the SOMS-2, therefore better suited to PC settings.

The ROC curve analysis for the FCSE yielded the same cut-off of the SSI (4 symptoms for men and 6 symptoms for women) reported by Escobar and colleagues [1], although the SOMS-2 asks for symptoms occurring only during the previous two years and not for lifetime symptoms. The cut-off of 4 symptoms is near the proposed 3 symptoms for Multisomatoform Disorder (MSD) [2], given these are current symptoms. At self-report, the R-SOMS-2 displayed seven false-positive cases and FCSE five. At interview those subjects stated that symptoms did not interfere at all with daily routines, this way loosing the severity threshold and becoming "negatives" when facing the interviewer. Using R-SOMS-2, we found 25 false-negatives, at self-report, seven of them overlapping with the 8 false-negatives at FCSE. Most of those 25 false-negatives used a medical disease they actually had as a kind of umbrella for other (unexplained) symptoms. At FCSE those symptoms turned to unexplained. As reported for false-positives, the clinical interview setting also seemed to interfere in the opposite way, leading some participants to enhance their complaints. This problem seems to be not exclusively explained by the relative instability of the individual system of causal attributions, since the observer presence affects the degree of severity of the complaints reported. Ten cases were considered probable "true" somatizers within the CS group, and among the false-negatives there were six cases considered as probable "true" somatizers. We think these patients will seldom screen positive using self-report measures of somatoform disorders or symptoms, since they need a thorough investigation involving their doctors in order to decide the nature of symptoms. This group of somatizers blurs the distinction between MUS and medically explained symptoms (MES). Along with the fact that MUS frequently prolong or are grafted on physical symptoms, they can add an explanation for the non relevance in distinguishing between MUS and MES when screening somatoform symptoms or disorders using self-report measures, as advocated by some authors [12,28]. In spite of that, the R-SOMS-2 presents a satisfactory sensitivity and good specificity for moderate somatization, being an adequate screening tool for referring primary care users for further specialized diagnosis. It is now possible to use in the PC Portuguese settings the adapted SOMS-2 as a checklist of DSM-IV and ICD-10 somatoform symptoms and the R-SOMS-2 as a shorter screener of possible cases. R-SOMS-2 is longer than two other screeners assessed in PC settings: the PHQ-15 (Patient Health Questionnaire) with 15 physical (explained or unexplained) symptoms [28], and the Othmer and DeSouza test with seven symptoms [16]. The first is considered to be a measure of severity of physical symptoms not yielding scores of self-report unexplained symptoms. The second, in a Spanish validation study, assessing its validity as a screener for Somatization Disorder [29] disclosed values of sensibility and specificity near those we obtained for moderate somatization (CS) with a cut-off of 4 symptoms: 88% and 78%, respectively, for a 3 symptom threshold.

The estimated prevalence of CS found in this study, 34.1%, is higher than the 14.0% for MSD, in Spanish PC units [5] and the median prevalence of 16.6% for the abridged SSI 4,6 criteria in a systematic review [30]. On one hand these studies used a more restrictive criteria, the SSI (4,6) and on the other hand women were over-represented in the sample used in our study. Nevertheless all PC users within the study period were invited to collaborate, irrespective of the motive why they came to the health centre, that may have been preventive procedures involving medical or nursing care (vaccinations, family planning a pregnancy routine checks) or consultations. This way it might be expected that persons were freer of mental distress, so the prevalence obtained is high, particularly when comparing with the Spanish study [5]. Another possible explanation being the fact that MSD is a current diagnosis and CS was considered during the previous two years. Indeed the prevalence of CS was higher in women as well as in participants with 4-8 years of formal education and presenting anxiety and depressive disorders. Another study in the southern Portugal disclosed a prevalence for DSM-IV Major Depression (as assessed by interview) of 13% [31] but only patients between 35-65 years with an appointment with their doctor were eligible. The more restrictive inclusion criteria (patients waiting for an appointment with the doctor) were expected to display a higher prevalence of depression as compared to the 19.2% found in this study. Gusmão *et a*l [32] in a review article reported a prevalence of 31.6% for depression (12% for clinical and 19.6% for sub-threshold depression) in a study carried out twenty years ago, during which 927 consecutive participants from Portuguese PC centres were interviewed [31]. A recent European study [33] displayed a prevalence for Major Depression in Portugal of 17.8% for women and 6.5% for men. In the same setting, we found 21.8% and 11.6%, respectively. Portuguese research on SFD and syndromes is lacking but we may conclude that there is a relative homogeneity in the prevalence of mental disorders in PC all over the country, specially for Major Depression.

The low response rate (18%) is a relevant limitation of the study, since the sample size is reduced and may not be representative of the PC population. Nevertheless the age distribution of participants was not significantly different from that of registered persons, but the over-representation of women may be responsible for the high prevalence found. Finally, we are aware that this validation study would have more strength if other PC units all over the country had been involved.

## Conclusion

The 29 items reduced version of the SOMS-2 showed to be a valid tool for detecting CS in primary care settings, specially whenever concomitant disorders such as anxiety and/or depression are present. Thus the R-SOMS-2 is a reliable referral tool for further specialized diagnosis.

## Competing interests

The authors declare that they have no competing interests.

## Authors' contributions

MCS, AB and CF conception and design; CF acquisition of data; MCS statistical analysis and interpretation of the data; MCS and CF drafting the manuscript; AB, MF and WR interpretation of data, critical revisions of the manuscript. All authors read and approved the final manuscript.

## Pre-publication history

The pre-publication history for this paper can be accessed here:

http://www.biomedcentral.com/1471-244X/10/34/prepub
